# A Seawater Salinity Sensor Based on Optimized Long Period Fiber Grating in the Dispersion Turning Point

**DOI:** 10.3390/s23094435

**Published:** 2023-04-30

**Authors:** Chao Du, Shuang Zhao, Qiuyu Wang, Bin Jia, Mingzhe Zhao, Li Zhang, Liqin Cui, Shizhe Chen, Xiao Deng

**Affiliations:** 1 College of Optoelectronics, Taiyuan University of Technology, Taiyuan 030024, China; duchao@tyut.edu.cn (C.D.); zhaoshuang0497@163.com (S.Z.); wangqiuyu1234@link.tyut.edu.cn (Q.W.); jiabin0160@link.tyut.edu.cn (B.J.); 15110837983@163.com (M.Z.); zhang219li@126.com (L.Z.); cuiliqin09@163.com (L.C.); 2 College of Physics, Taiyuan University of Technology, Taiyuan 030024, China; 3 Institute of Oceanographic Instrumentation, Qilu University of Technology (Shandong Academy of Sciences), Qingdao 266001, China; chensz@qlu.edu.cn

**Keywords:** fiber grating, salinity measurement, high sensitivity, etching cladding

## Abstract

Variations of seawater salinity often cause ocean internal waves, water masses and stratification, which affect the stability of the ocean environment. Therefore, the study of seawater salinity is significant for the prediction of changes in the ocean environment. However, existing methods for measuring seawater salinity generally have the disadvantages of low sensitivity and low accuracy. In this work, we proposed a seawater salinity sensor based on long period fiber grating (LPFG) in the dispersion turning point (DTP), which has demonstrated the possibility to fabricate LPFG with a shorter grating period by CO_2_ laser in a thin single mode fiber (SMF) of 80 μm cladding diameter without etching. For obtaining higher sensitivity that could meet the measurement requirement in practice, the proposed sensor was optimized by combining etching cladding and DTP. After the LPFG working near DTP was fabricated by a CO_2_ laser, the cladding diameter was reduced to 57.14 μm for making cladding mode LP_1,7_ work near DTP by hydrofluoric acid (HF) solutions. The experimental results have demonstrated that a sensitivity of 0.571 nm/‰ can be achieved when the salinity increases from 5.001‰ to 39.996‰, and the sensor shows good repeatability and stability. Based on its excellent performance, the optimized LPFG is a prospective sensor to monitor seawater salinity in real time. Meanwhile, a low-cost way was provided to make LPFG work near DTP instead of ultraviolet exposure and femtosecond laser writing.

## 1. Introduction

The ocean is rich in water, mineral and biological resources, which is greatly important to the survival and development of mankind. In particular, salinity is a key parameter to monitor the ocean environment and climate [1]. The variations of seawater salinity can cause some phenomena, such as ocean internal waves, water masses and stratification, which have an important impact on the ocean environment and can even threaten the safe operation of underwater military equipment, such as submarines [2,3,4]. Therefore, it is essential to explore an accurate, real-time and highly sensitive method for measuring seawater salinity to ensure the stability of the ocean environment. In recent years, the methods to measure seawater salinity have been developed rapidly [5,6,7].

Universally, the seawater salinity is measured according to electrical conductivity, which is based on the relationship between the ion content and conductivity [8]. The electrical conductivity of seawater is related to temperature, pressure and salinity. Therefore, the electrical conductivity is only determined by salinity under the same temperature and pressure. However, the accuracy of the electrical conductivity method is susceptible to electric interference. Moreover, the seawater salinity can be measured on a large scale by microwave remote sensing [9,10]. The brightness temperature data of the sea surface are measured by the microwave radiometer on a remote sensing satellite to further obtain the sea surface salinity. However, the microwave remote sensing method can only measure the salinity of the sea surface with low accuracy, which makes it unsuitable for high-precision salinity measurement. At present, the research on optical fiber sensors measuring seawater salinity has attracted widespread attention from domestic and international scholars due to the advantages of fast response, anti-electromagnetic interference, small size and corrosion resistance [11].

The optical fiber sensors mainly depend on the relationship between the refractive index (RI) and salinity to measure the salinity of seawater [12]. The salinity of seawater generally has the range of 5–40‰ (with the RI range of 1.333–1.339). Based on different principles, optical fiber salinity sensors mainly include fiber interferometer [13,14,15], fiber surface plasmon resonance (SPR) [16,17,18], fiber Bragg grating (FBG) [19,20] and long-period fiber grating (LPFG) [21,22,23]. The fiber interference is generally made by splicing procedures, which makes the sensor lossy and the structure fragile. Although the fiber SPR has higher salinity sensitivity, the wide bandwidth makes its demodulation accuracy low. The FBG has high demodulation accuracy, but the bare FBG is insensitive to RI. Compared with other fiber-sensing structures, LPFG is more suitable for seawater salinity measurement due to the advantages of RI sensitivity and easy integration. However, the sensitivity of conventional LPFG still cannot meet the requirements of salinity measurement. Presently, the RI sensitivity of LPFG can be improved by three approaches, such as etching cladding, making LPFG work within the mode transition (MT) or dispersion turning point (DTP) region [24]. However, the method of coating high RI nanofilms is more complicated, and high precision is demanded for the thickness and evenness of nanofilm, so reducing appropriate cladding combined with making LPFG work near DTP is a more effective way to improve sensitivity.

There are many methods to fabricate LPFG, such as femtosecond lasers writing [25,26], arc inducing [27,28], ultraviolet (UV) exposure [29,30,31] and CO_2_ laser writing [32,33], in which UV exposure with an amplitude mask is the most widely used and effective method to fabricate LPFG working near DTP. In 2017, Janczuk-Richter et al. proposed a LPFG virus sensor based on hydrogen-loaded SMF-28 fiber by KrF excimer laser [29]. Afterwards, Šmietana et al. proposed a RI sensor based on LPFG working near DTP with Al_2_O_3_-nanocoated, which was fabricated by UV exposure [30]. In 2018, Del Villar et al. proposed an optimized LPFG strain sensor working near DTP, which was fabricated by the point-by-point inscription technique by KrF excimer laser [31]. However, the UV laser is too expensive to make it possible for most people to fabricate LPFG working near DTP. In addition, amplitude mask writing has higher requirements for the photosensitivity of fiber, and the LPFG is easily erased. Compared to a UV laser, a CO_2_ laser has the advantages of low cost, high reliability and applicable for any fiber types. The LPFG based on CO_2_ laser technology has attracted more and more attention, but few people make LPFG work near DTP by CO_2_ laser.

In order to explore a low-cost, easily fabricated and highly sensitive salinity sensor, we proposed a CO_2_-laser-fabricated LPFG working near DTP with a period of 115 μm, which was induced in thin SMF. The LPFG has good sensing characteristics and greater attenuation loss peaks. Furthermore, the RI sensitivity of LPFG was further improved by making it work near DTP and etching cladding. On the basis of this, an LPFG whose resonance wavelengths were operated near DTP was designed by adjusting the grating period to 110 μm. Then, the cladding of LPFG was etched to 57.14 μm by HF solution, and the coupled cladding mode worked near DTP. The salinity sensitivity can reach 0.571 nm/‰ when the salinity increases from 5.001‰ to 39.996‰. The combination of the above two methods not only significantly improves its sensitivity, but also ensures the mechanical strength of LPFG.

The contents of this work are as follows: In Section 2, the related work of seawater salinity measurements is outlined. In Section 3, the coupled mode theory and RI sensing performance of LPFG are introduced, which provide the theoretical basis for the experiments in this work. Furthermore, the fabrication method of LPFG and materials are introduced. In Section 4, the simulation model of LPFG is built, and the experimental results are discussed based on theoretical results. Finally, the repeatability and stability of LPFG seawater salinity sensors are investigated. In Section 5, a comprehensive conclusion is given.

## 2. Related Work

In this section, different measurement methods for seawater salinity are shown, including electrical conductivity measurement, microwave remote sensing and optical fiber sensors, in which an optical fiber sensors’ section is divided into four parts, such as fiber interferometer, SPR, FBG and LPFG.

### 2.1. Electrical Conductivity Measurement

In 2015, Pawlowicz et al. reviewed the history of seawater salinity measurement using electrical conductivity and analyzed the problems with current measurement methods [34]. Furthermore, future methods for measuring seawater salinity were discussed in the background of recent seawater standard TEOS-10. In 2018, Schmidt et al. proposed a new relationship between density and salinity by correcting for the density of uniform isotopes and chemical composition [5]. The proposed new relationship of density–salinity can be used to verify the constant composition of standard seawater by measuring conventional density. In 2022, Parra et al. proposed a low-cost multi-parameter detector [35]. The sensors integrated in the wireless network are deployed in coastal areas, and the salinity data can be monitored in real time. The measurement results show the coefficient of the salinity correction model is 0.9, and the mean absolute error is 0.74 g/L.

### 2.2. Microwave Remote Sensing

In 2019, Dinnat et al. proposed a comparison of long-term global spatial distributions, a set of regions of interest and the temporal variability of statistical distributions [9]. They found that the discrepancies of salinity measurements in the sea surface were mainly caused by dielectric constant models, atmospheric corrections and sea surface temperature auxiliary products. In 2022, Demir et al. carried out an inversion study of sea ice and salinity by using a 0.5–2 Ghz radiometer [7]. They found that the higher sensitivity of first-year sea ice thicknesses can be achieved in the 0.5–1.5 m range, and the thickness and salinity can be retrieved simultaneously compared to a 1.4 Ghz microwave radiometer. Afterwards, Le Vine et al. studied the effects of temperature and wind speed on the accuracy for salinity remote sensing in the frequency range of 0.3–3.0 GHz [36]. They found that lower frequencies and temperatures were more beneficial for salinity measurements, but had the potential to increase errors.

### 2.3. Optical Fiber Sensors

#### 2.3.1. Fiber Interferometer

In 2017, Wang et al. proposed and demonstrated an ultrasensitive, multimode interferometer salinity sensor based on Panda-microfiber [13]. A section of multimode fiber was inserted into a conventional SMF to achieve higher-order modes, and a high sensitivity of 2938.16 pm/‰ was achieved. In 2021, Lin et al. proposed a high-sensitivity seawater salinity sensor based on the Mach–Zehnder structure [14]. The fiber with double-side holes was etched into an open-cavity structure, and a salinity sensitivity of 2 nm/‰ could be achieved when the cavity length was about 100 μm. In 2022, Zhao et al. proposed a hybrid fiber optic sensor for simultaneous measurement of seawater temperature and salinity [37]. In particular, a section of hollow-core fiber with a U-shaped groove was connected between 2 SMFs to measure seawater salinity, and a salinity sensitivity of 0.244 nm/‰ could be achieved.

#### 2.3.2. SPR

In 2017, Zhao et al. proposed a seawater salinity sensor based on a C-shaped optical fiber structure [38]. Based on the SPR principle, a gold film was coated on the fiber surface to improve its sensitivity, and a salinity sensitivity of 1.402 nm/‰ could be achieved. In 2020, Siyu et al. proposed a SPR sensor for measuring seawater salinity [17]. A gold film was coated on the surface of hollow-core fiber to excite the SPR effect for improving the sensitivity. The experimental results showed that the sensor had a salinity sensitivity of 0.3769 nm/‰. In 2021, Yang et al. proposed an SPR salinity sensor based on an exposed fiber core structure [39]. Indium tin oxide and gold films were sequentially coated on the exposed area of fiber core to improve the sensitivity. The sensor’s RI sensitivity was up to 3000 nm/RIU.

#### 2.3.3. FBG

In 2019, Sun et al. proposed a FBG salinity sensor coated with lamellar polyimide to measure the salinity in a gravel aquifer [19]. The results showed that the sensitivity of 0.0358 nm/% could be reached, and the proposed sensor had good consistency and repeatability. Afterwards, Kumari et al. proposed a highly accurate and fast responsive Nuttall apodized FBG sensor coated with hygroscopic polymer to measure seawater salinity [20]. The sensitivity was improved to 0.0026 nm/PSU by coating Polyimide on the FBG surface. Moreover, the measurement accuracy was improved by using the Nuttall apodization function, and the average error was reduced to 0.2015 PSU. In 2021, Raghuwanshi et al. proposed an etched FBG RI sensor based on an α-power RI profile fiber [40]. The cladding diameter of FBG was etched to different thicknesses to improve its sensitivity, and the maximum sensitivity of 35 nm/RIU could be achieved.

#### 2.3.4. LPFG

In 2015, Del Villar proposed an optimized LPFG by combining the reducing cladding diameter, DTP and MT, which theoretically achieved a sensitivity of 1.43 × 10^5^ nm/RIU [24]. In 2017, Yang et al. proposed a conventional LPFG salinity sensor, and its sensitivity could reach 36 nm/M (0.615 nm/‰) by coating with polyelectrolyte multilayers [21]. Afterwards, Yang et al. once again proposed a salinity sensor based on LPFG coated with hydrogel, and its salinity sensitivity was 0.1255 nm/‰ [22]. In 2021, Dey et al. designed a LPFG RI sensor whose low order cladding modes had DTP at longer wavelengths by reducing the cladding diameter [41]. Although a sensitivity of 8751 nm/RIU for cladding mode LP_0,2_ was realized, the etched cladding diameter of 21.87 μm was so thin that it was easy to be broken during the measurement process in practice. In 2021, Viveiros et al. proposed a LPFG working in DTP, and the RI sensitivity could attain 3151.7 nm/RIU by coating with TiO_2_ when the surrounding refractive index (SRI) increased from 1.33 to 1.37 [42].

Although significant progress has been made in seawater salinity measurement technology, there are still various problems that prevent accurate, real-time and highly sensitive measurement of seawater salinity. For example, electric interference has a large impact on the electrical conductivity measurement. In addition, microwave remote sensing is limited by the measurement resolution to achieve high accuracy measurement. For optical fiber sensors, the demodulation accuracy of the fiber interferometer and SPR is low, and the sensitivity of FBG is low. Compared with other measurement methods of seawater salinity, LPFG has the advantage of sensitivity to SRI, but the conventional LPFG also cannot meet the measurement requirement. As is well known, reducing the cladding diameter and making the LPFG work near DTP are effective methods to improve the sensitivity of LPFG. Therefore, the optimized LPFG at DTP was proposed to obtain a seawater salinity sensor with higher sensitivity.

## 3. Materials and Methods

The basic principle of LPFG is coupled mode theory, in which the cladding mode will be coupled with the core mode when they meet the phase match condition [43,44]. The phase match condition of LPFG is

(1) $$\lambda =\left(n_{eff}^{co}-n_{eff,m}^{cl}\right)\Lambda$$

in which λ is the resonance wavelength; Λ is the grating period; $n_{eff,m}^{cl}$ and $n_{eff}^{co}$ are effective RIs of the *m*th cladding and core mode, respectively. When the grating parameters are determined, the relationship between the LPFG resonance wavelength shift and SRI is approximately expressed as [45].

(2) $$\frac{d\lambda }{dn_{3}}=\lambda \cdot \gamma \cdot \Gamma$$

in which $n_{3}$ is the SRI. γ is the waveguide dispersion and is expressed as

(3) $$\gamma =\frac{\frac{d\lambda }{d\Lambda }}{n_{eff}^{co}-n_{eff,m}^{cl}}$$

Γ describes the SRI dependences of the waveguide dispersion and is defined by

(4) $$\Gamma =-\frac{U_{m}^{2}\lambda^{3}n_{3}}{8\pi a_{2}^{3}n_{2}(n_{eff}^{co}-n_{eff,m}^{cl}){\left(n_{2}^{2}-n_{3}^{2}\right)}^{3/2}}$$

in which $n_{2}$ and $a_{2}$ are the RI and radius of cladding, and $U_{m}^{2}$ is the *m*th root of the zeroth-order Bessel function of the first kind. It can be found that the effective solution for increasing sensitivity is the etching cladding. With the decrease of the cladding diameter, the order of the cladding mode gradually approaches that of the next lower-order cladding mode, and the interaction between the cladding mode and the surrounding medium is enhanced, which leads LPFG to sense SRI more sensitively. Furthermore, the RI sensitivity is relevant to the order of the cladding mode, and it will increase with the higher order of the cladding mode. In particular, there is a DTP on the phase match curve (PMC) between the core mode and the higher-order cladding mode in the near-infrared wavelength region, and the sensitivity of LPFG will reach a maximum at DTP [45,46].

**Figure 1:**
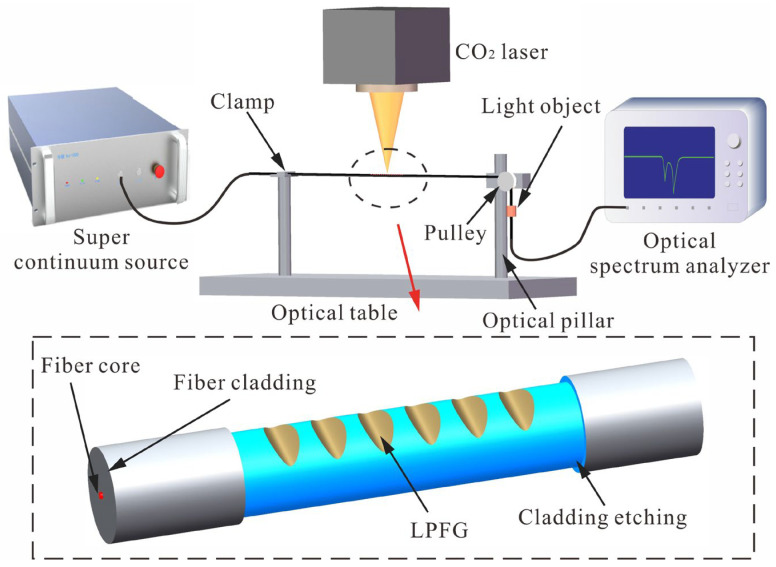
Experimental device of CO_2_ laser fabricating LPFG.

The formation mechanism of LPFG fabricated by a CO_2_ laser is relatively complex. It is generally believed that there are mainly stress release, density variation, melt deformation and local etching, which not only change the RI of fiber, but also have a large influence on the structure of fiber. Therefore, the coupled local-mode theory is more suitable for the LPFG fabricated by a CO_2_ laser, whose resonance wavelength can be approximately expressed as [47].

(5) $$N\lambda =\left(n_{eff}^{co}-n_{eff}^{cl}\right)\Lambda$$

in which N is the harmonic component. The process of fabricating LPFG with a CO_2_ laser is shown in Figure 1. The CO_2_ laser with a power of 10 W (CO_2_-H10C, Han’s laser) is located directly above the fiber to ensure that the spot of the laser can be focused on the fiber. In particular, the grating period, depth and width are the most important parameters for the grating formation, which are related to the fabrication parameters. In order to simplify the fabrication process, the period and laser power are generally changed to fabricate the desired LPFG, and other fabrication parameters are kept unchanged, which is shown in Table 1. Moreover, the super continuous light source (SC-5, YSL photonics) with the output power of about 500 mW is connected with an optical spectrum analyzer (OSA: AQ6370D, Yokogawa) by optical fiber and monitoring the transmission spectra in real time to ensure the quality of LPFG.

**Figure 2:**
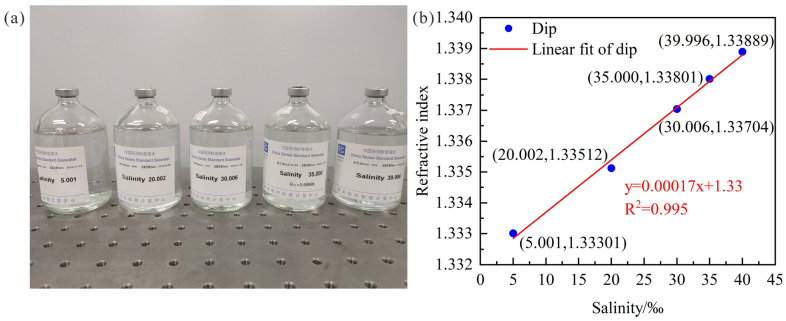
(**a**) Standard seawater solutions; (**b**) Relationship between RI and salinity.

As shown in Figure 2, the China series standard seawater solutions (GBW(E)130011, National Center of Ocean Standards and Metrology) composed by treated natural seawater were measured by an Abel refractometer (WAY-3S, INESA), and the RI range is 1.33301 to 1.33889 when the seawater salinity increases from 5.001‰ to 39.996‰. Therefore, the RI sensitivities of LPFG are approximated by wavelength variations when SRI increases from 1.33 to 1.34 during simulation. In addition, the variation of seawater salinity was replaced by RI to compare the theoretical and experimental results more clearly. Finally, the data was analyzed by OriginPro 2018C from OriginLab corporation.

## 4. Results and Discussion

**Figure 3:**
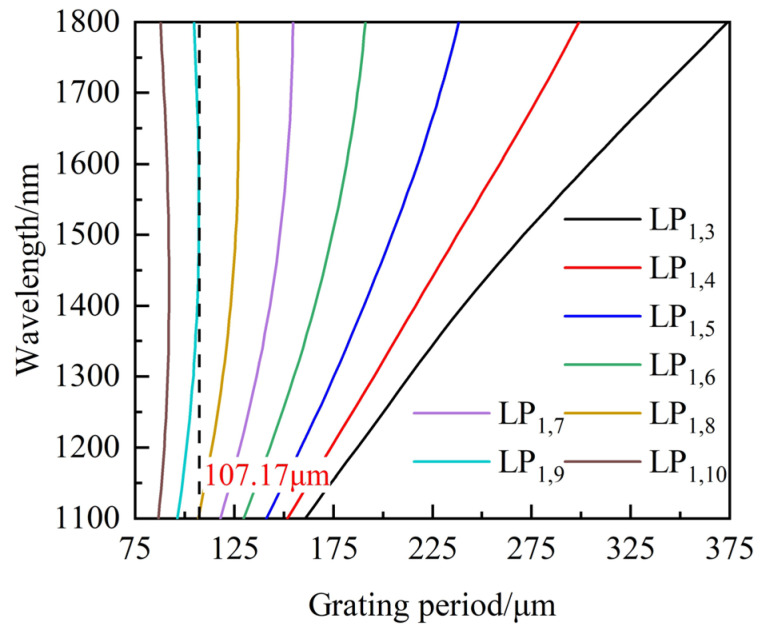
PMCs of LPFG based on thin SMF.

The designed seawater salinity sensor relies on LPFG sensing characteristics, so the SRI sensitivity is the crucial performance indicator. The model of LPFG for simulation is built on the basis of thin SMF (SMF13-2(21111)-3B, the 46th Institute of China Electronics Technology Group Corporation), whose core diameter and RI are 5.2 μm and 1.471, respectively, and the cladding diameter and RI are 80 μm and 1.46, respectively (with an incident wavelength of 1550 nm). The core RI is periodically modulated by CO_2_ laser, so its coupling mode of LPFG is asymmetric [48]. As is well known, the dispersion characteristics of SMF are related to the material RI and structure parameters of the core and cladding, respectively. In order to optimize the fabrication parameters to ensure the cladding mode works in DTP at a specific wavelength range, the PMCs based on thin SMF are plotted in Figure 3, in which the SRI is 1. It can be found that the DTP of LPFG coupled by the cladding mode LP_1,9_ appears when the grating period is less than 107.17 μm, and there are 2 resonance wavelengths for the same grating period near DTP.

### 4.1. Conventional LPFG

**Figure 4:**
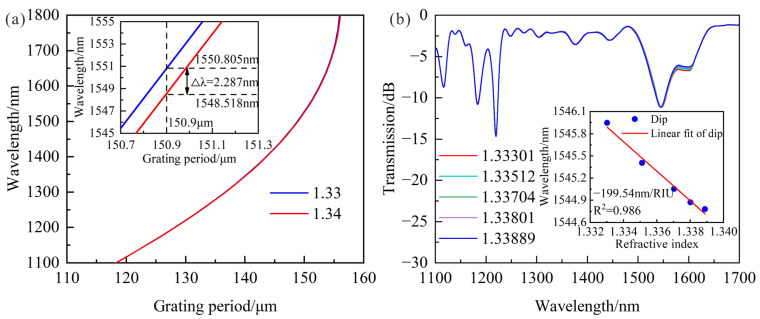
(**a**) Theoretical wavelength shifts of LPFG with the period of 162 μm when SRI changes from 1.33 to 1.34; (**b**) Transmission spectra and the relationships between resonance wavelength and RI variations when SRI increases from 1.33301 to 1.33889.

As shown in Figure 3, the order of the cladding mode is higher in thin SMF when the designed LPFG works near DTP when the wavelength range is from 1100 nm to 1800 nm. Therefore, the cladding mode of LP_1,7_ was firstly chosen to improve the RI sensitivity by etching cladding to make LPFG work in DTP. The PMCs of LPFG with the cladding mode LP_1,7_ and an 80 μm cladding diameter are shown in Figure 4a, and there are no DTPs when the wavelength range is from 1100 nm to 1800 nm. It can be found that the wavelength decreases by 2.287 nm when SRI increases from 1.33 to 1.34. The LPFG with a grating period of 162 μm was fabricated by a CO_2_ laser with the laser power of 20% total power, and the experimental result is shown in Figure 4b, and the RI sensitivity is −199.54 nm/RIU, which is in agreement with the theoretical result.

**Figure 5:**
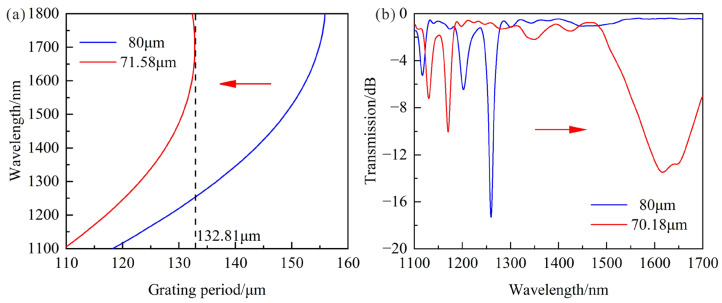
(**a**) PMCs of LPFG with LP_1,7_ cladding mode when cladding diameter decreases to 71.58 μm and the SRI is 1; (**b**) Transmission spectra when cladding diameter decreases to 70.18 μm and the SRI is 1.

As shown in Figure 5a, the lower-order cladding mode could work near DTP by etching cladding. It can be found that the DTP appears when the grating period and cladding diameter are less than 132.81 μm and 71.58 μm, respectively. In fact, the LPFG with the period of 145 μm was fabricated by a CO_2_ laser with the laser power of 20% total power. Then, the cladding of LPFG was etched to 70.18 μm by 40% and 10% hydrofluoric acid (HF) solutions, respectively. As shown in Figure 5b, the shifts of the transmission spectra were monitored in real time by OSA. The resonance wavelength moves towards a longer wavelength, and the DTP appears when the wavelength range is from 1100 nm to 1700 nm as the cladding diameter decreases.

**Figure 6:**
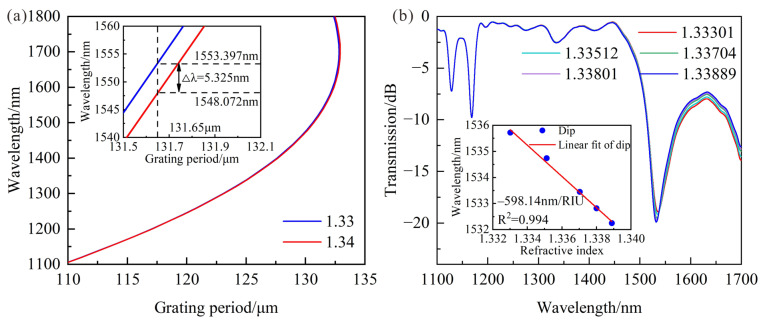
(**a**) PMCs of LPFG with 71.58 μm cladding diameter when the SRI changes from 1.33 to 1.34; (**b**) Transmission spectra of LPFG with 70.18 μm cladding diameter and LP_1,7_ cladding mode, and the relationships between resonance wavelength and RI variations when SRI increases from 1.33301 to 1.33889.

As is well known, the resonance wavelength will move away from the DTP as SRI increases. The PMCs of LPFG with a 71.58 μm cladding diameter are shown in Figure 6a, and there is only a shorter resonance wavelength when the wavelength range is from 1100 nm to 1700 nm. In addition, the shorter wavelength decreases by 5.325 nm when SRI increases from 1.33 to 1.34. The spectral responses of LPFG are shown in Figure 6b, and the longer resonance wavelength is beyond the monitoring range of OSA. It can be found that the RI sensitivity of the LPFG shorter resonance wavelength is −598.14 nm/RIU, which was enhanced twice after reducing the cladding diameters. Compared with unetched LPFG, the RI sensitivity was improved, but it still could not meet the highly sensitive measurement requirements of the salinity.

### 4.2. LPFG Working near DTP

**Figure 7:**
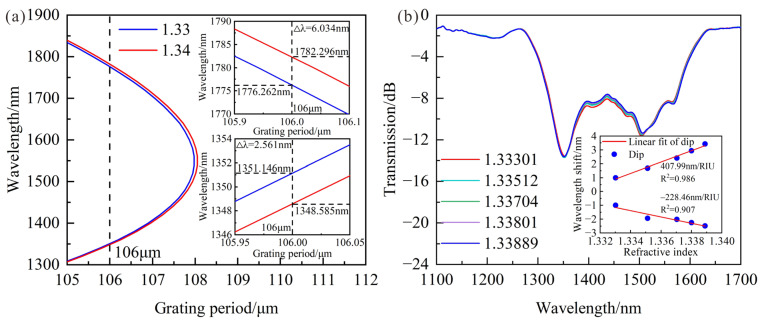
(**a**) Theoretical wavelength shifts of LPFG with 80 μm cladding diameter when SRI changes from 1.33 to 1.34; (**b**) Transmission spectra and the relationships between resonance wavelength and RI variations when SRI increases from 1.33301 to 1.33889.

In order to further improve the sensitivity of LPFG, a high-order cladding mode LP_1,9_ was chosen to make LPFG work near DTP. The PMCs of LPFG with an 80 μm cladding diameter are shown in Figure 7a, and there are 2 resonance wavelengths when the grating period is less than 108 μm. It can be found the shorter wavelength decreases by 2.561 nm and the longer wavelength increases 6.031 nm when SRI increases from 1.33 to 1.34. In accordance with the technical manual of the CO_2_ laser, it is known that the surface of the fiber is hit by a laser spot with a size of 100 μm, and the precision of the period is about 1 μm. Therefore, the LPFG working near DTP can be theoretically fabricated by a CO_2_ laser without etching.

**Figure 8:**
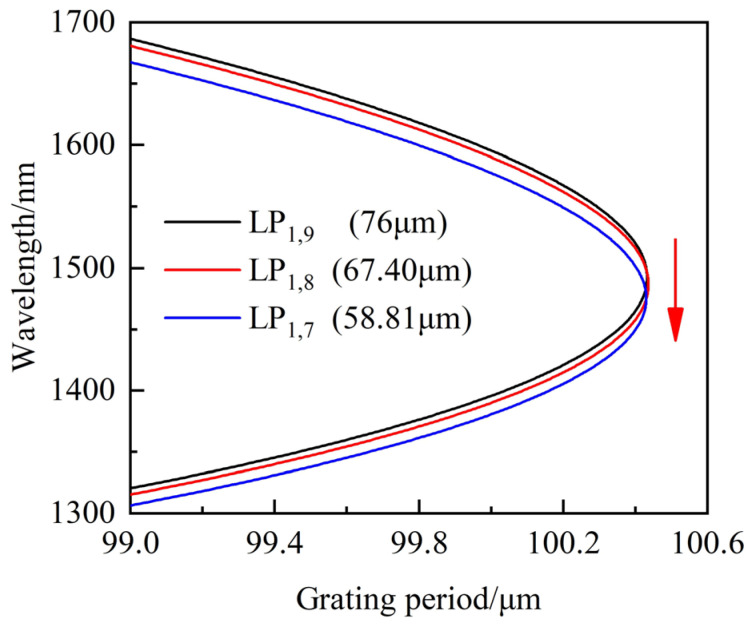
PMCs of LPFG under different cladding modes and diameters.

In fact, the LPFG with cladding mode LP_1,9_ works near DTP when the period is 115 μm. The spectral responses of LPFG are shown in Figure 7b, in which SRI increases from 1.33301 to 1.33889. It can be found that the RI sensitivities at shorter and longer resonance wavelengths are −228.6 nm/RIU and 407.99 nm/RIU, which is higher than conventional LPFG. However, the LPFG with improved RI sensitivity still cannot meet the highly sensitive requirements of salinity measurement. Furthermore, the cladding of LPFG working near DTP should be etched to improve its sensitivity. The PMCs of LPFG under different cladding modes and diameters are shown in Figure 8, in which the SRI is 1. The DTP moves towards a shorter wavelength direction, and the shape of PMCs for cladding modes LP_1,9_, LP_1,8_ and LP_1,7_ remain basically unchanged when the cladding diameters are reduced from 80 μm to 76 μm, 67.4 μm and 58.81 μm.

**Figure 9:**
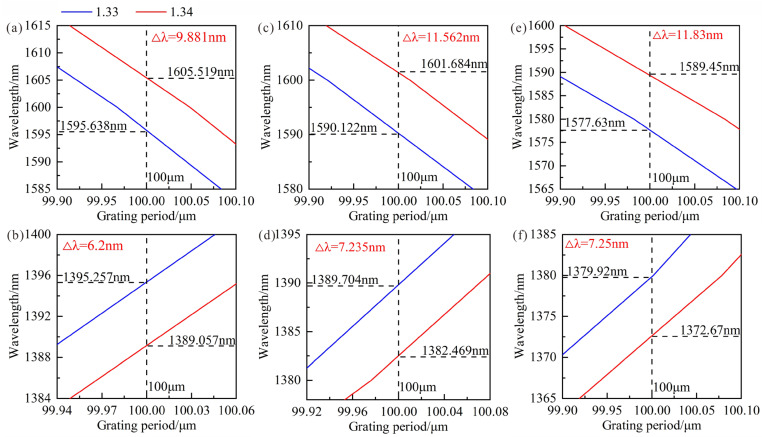
Theoretical wavelength shifts of LPFG under different cladding modes and diameters when SRI changes from 1.33 to 1.34: (**a**,**b**) LP_1,9_ and 76 μm; (**c**,**d**) LP_1,8_ and 67.4 μm; (**e**,**f**) LP_1,7_ and 58.81 μm.

The theoretical results of RI sensitivity are shown in Figure 9, when the SRI increases from 1.33 to 1.34. It can be found that the shorter wavelength of LPFG with the cladding modes LP_1,9_, LP_1,8_ and LP_1,7_ decrease 6.2 nm, 7.235 nm and 7.25 nm, and the longer wavelength increases 9.881 nm, 11.562 nm and 11.83 nm. As the cladding diameter decreases, the obtained average RI sensitivities at a shorter wavelength are −620 nm/RIU, −723.5 nm/RIU and −725 nm/RIU, and the RI sensitivities at a longer wavelength are 988.1 nm/RIU, 1156.2 nm/RIU and 1183 nm/RIU, respectively. Therefore, reducing the appropriate cladding diameter of LPFG working near DTP is an effective way to overcome the contradiction between sensitivity and mechanical strength.

**Figure 10:**
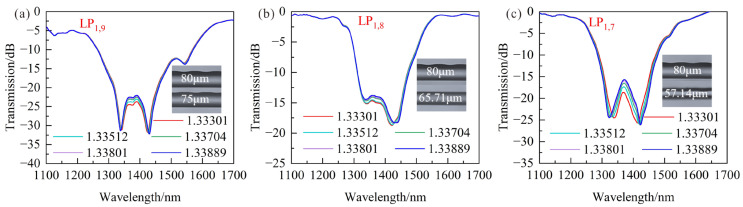
Transmission spectra of LPFG with the period of 110 μm under different cladding modes and diameters when SRI increases from 1.33301 to 1.33889: (**a**) LP_1,9_ and 75 μm; (**b**) LP_1,8_ and 65.71 μm; (**c**) LP_1,7_ and 57.14 μm.

In order to get a higher RI sensitivity, the LPFG with a period of 110 μm was fabricated by a CO_2_ laser with the laser power of 20% total power. Furthermore, the number of scanning cycles is 1 to obtain a high-quality LPFG due to about 1 μm precision of the grating period. Then, the LPFG claddings were etched by 40% and 10% HF solutions, and the 10% HF solution was used to achieve a higher-precision etching process when the two resonance wavelengths were closer. The transmission spectra of LPFG under different cladding diameters and modes are shown in Figure 10, and the LPFGs have a deep modulation loss of 25 dB when the SRI increases from 1.33301 to 1.33889., which makes them more suitable for applications in salinity measurement.

**Figure 11:**
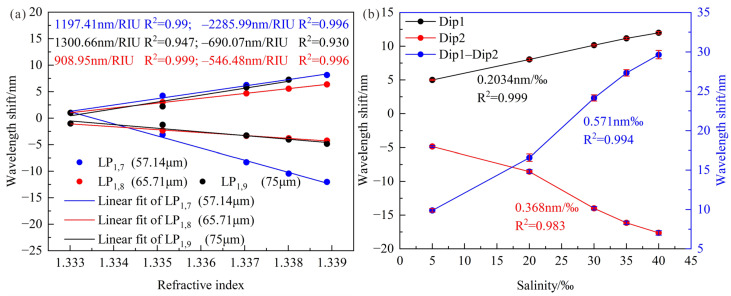
(**a**) Relationships between SRI and wavelength shifts for LPFG under different cladding modes and diameters; (**b**) Relationships between salinity and wavelength shifts for LPFG with cladding diameter of 57.14 μm and cladding mode LP_1,7_.

The relationships between SRI and the wavelength shifts for different cladding modes and diameters are shown in Figure 11a, in which the SRI increases from 1.33301 to 1.33889. It can be found that the average RI sensitivities of LPFG whose cladding diameters are 75 μm, 65.71 μm and 57.14 μm at shorter wavelengths are −546.48 nm/RIU, −690.07 nm/RIU and −2285.99 nm/RIU, respectively. In particular, the sensitivity is more sensitive to variations of the grating period at DTP, which makes the sensitivity of the shorter wavelength change a lot when the grating period changes 1 μm. Moreover, the RI sensitivities at longer wavelengths are 908.95 nm/RIU, 1300.66 nm/RIU and 1197.41 nm/RIU, which are well agreed with the theoretical results. As shown in Figure 11a, the high sensitivity of 3483.4 nm/RIU could be obtained by calculating the wavelength differences between two resonance peaks when the cladding diameters were reduced to 57.14 μm. Therefore, the LPFG with a cladding diameter of 57.14 μm and a cladding mode LP_1,7_ can be used to measure seawater salinity due to its excellent performance.

**Figure 12:**
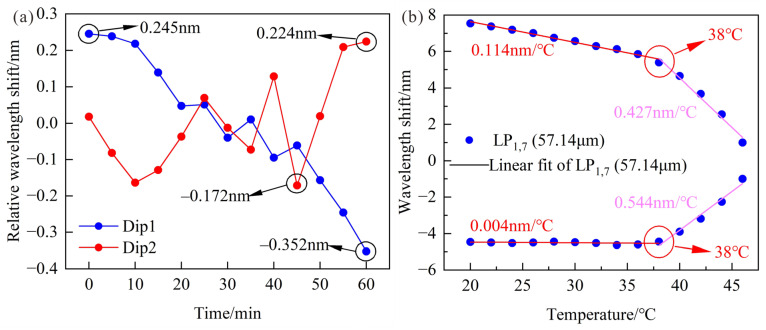
(**a**) Relative wavelength shifts of the optimized LPFG within 60 min in water; (**b**) Relationships between wavelength shifts and temperature.

Based on the preliminary RI experiment, the LPFG was maintained straight and placed in standard seawater solutions with a salinity range of 5.001–39.996‰ to investigate the salinity-sensing performance. The relationships between salinity and wavelength shifts are shown in Figure 11b, and the high sensitivity of 0.571 nm/‰ could be obtained by calculating the wavelength differences between 2 resonance peaks, and the LPFG has a good linearity of 0.994. In addition, 3 cycle experiments were designed to demonstrate the repeatability, and the average standard deviation of 0.403 nm caused a salinity error of 0.7‰. In order to certify the stability of the sensor, the sensor was immersed in water with a salinity of 0‰ for 60 min and the temperature maintained at about 19.8 °C. The variations of the resonance wavelength with time are shown in Figure 12a, and the maximum fluctuation of the resonance wavelength is 0.352 nm, and the salinity error is 0.62‰, which is acceptable.

To further explore the cause of wavelength fluctuations, the LPFG was immersed in water solutions with a temperature range of 20–46 °C to investigate the temperature sensing performance. The relationships between wavelength shifts and temperature are shown in Figure 12b, and there is a significant difference of temperature sensitivity at 38 °C. In particular, it can be found that the temperature sensitivities of longer and shorter resonance wavelengths are 0.114 nm/°C and 0.004 nm/°C in the temperature range of 20–38 °C, which results in little wavelength fluctuations. Therefore, the fluctuations of resonance wavelength may be caused by noise or unavoidable handling during the experiment. It is necessary to seek better ways to remove noise or improve the RI sensitivity of LPFG for reducing the influence of wavelength fluctuations.

### 4.3. Discussion

Comparisons between other LPFG salinity or RI sensors and this work are shown in Table 2; the LPFGs work near DTP, except for the LPFGs in [21,22]. In theory, the sensitivity of 1.43 × 10^5^ nm/RIU can be achieved by combining a reducing cladding diameter, DTP and MT, but the RI range (1.33–1.331) is beyond the RI range of the seawater salinity. In practice, the LPFG fabricated in this work has a higher sensitivity compared to the other LPFG fabricated by a CO_2_ laser in [21]. Furthermore, it can be found that the LPFG in this work has a similarly high RI sensitivity compared to the LPFGs fabricated by femtosecond and UV laser. In particular, the RI sensitivity of the LPFG in this work is higher than the arc-induced LPFG in [27]. Finally, the RI sensitivity of the LPFG fabricated in this work is similar to that of the LPFG coating with thin-film, which avoids the complex coating deposition. In summary, the LPFG fabricated in this work has significant advantages in salinity measurement. In addition, the LPFG salinity sensor was fabricated in a low-cost way, which also significantly reduced the fabrication cost.

## 5. Conclusions

A LPFG seawater salinity sensor has been proposed, which has significant advantages in salinity measurement. The LPFG worked near DTP and was economically fabricated by a CO_2_ laser without any special processing, which could replace femtosecond and UV lasers. Then, the LPFG with a reduced cladding diameter of 57.14 μm has designed to make the cladding mode LP_1,7_ work near DTP, and a salinity sensitivity of 0.571 nm/‰ could be obtained in the salinity range of 5.001–39.996‰. In summary, the LPFG seawater salinity sensor has the advantages of high sensitivity, low loss, simple fabrication and stability, which allows it to be more widely used in seawater salinity measurement.

Even so, there is still great potential for improving its performance in future research work. Firstly, the fabrication method of a CO_2_ laser will be improved to fabricate a high-quality dual-peak resonance LPFG. In addition, a comprehensive method will be proposed to obtain higher-sensitivity LPFGs by combining reducing cladding diameters and making LPFG work near MT and DTP regions. Finally, the effects of the seawater environment should be considered in practical seawater salinity measurement. Therefore, the package structure of the proposed seawater salinity sensor will be designed to allow it to be exposed to seawater, while reducing the influence of seawater on the sensor probe.

## Figures and Tables

**Table 1:** Parameters of LPFG fabrication in this work.

Fabrication Parameter	Value
Marking speed	30 mm/s
Air jump speed	1000 mm/s
Q-frequency	20 kHz
Turn-on delay time	100 μs
Turn-off delay time	100 μs
Jump delay time	300 μs
Corner delay time	10 μs

**Table 2:** Comparisons between other LPFG salinity or RI sensors and this work.

Fabrication Process	Cladding Diameter (μm)	Sensitivity (nm/‰)	Sensitivity (nm/RIU)	Range	Reference
1. CO_2_ laser writing 2. Coating with polyelectrolyte	125	0.612	~	29.25–46.8‰	[21]
1. CO_2_ laser writing 2. Coating with hydrogel	125	0.1255	~	22.8–44.7‰	[22]
1. Femtosecond laser writing 2. Coating with TiO_2_	125	~	3157.7	1.33–1.37	[42]
			4294.5	1.33–1.34	
1. KrF excimer laser with amplitude mask writing 2. HF solutions etching 3. Coating with TiO_2_	Less than 125	~	6200	Close to 1.3400	[49]
			4300	1.36–1.41	
1. Doubled argon laser writing 2. HF solutions etching	71.75	~	1343	1.353–1.398	[50]
	32.5		8734		
Theoretical work: 1. HF solutions etching	29.24	~	3750	1.33–1.35	[24]
1. HF solutions etching 2. Coating with thin-film	34.8	~	143,000	1.33–1.331	
Arc inducing	125	~	720	1.33–1.42	[27]
1. CO_2_ laser writing 2. HF solutions etching	57.14	0.571	3483.4	5.001–39.996‰ (1.33301–1.33889)	This work

## Data Availability

The data presented in this study are available on request from the corresponding author.

## References

[1] Sohail T., Zika J.D., Irving D.B., Church J.A. (2022). Observed poleward freshwater transport since 1970. Nature.

[2] Kim H., Son Y.B., Jeong J.Y., Jo Y.H. (2018). Comparison of internal waves in various ocean fields around the korean peninsula. J. Coast. Res..

[3] Silvy Y., Guilyardi E., Sallee J.B., Durack P.J. (2020). Human-induced changes to the global ocean water masses and their time of emergence. Nat. Clim. Chang..

[4] Li G.C., Cheng L.J., Zhu J., Trenberth K.E., Mann M.E., Abraham J.P. (2020). Increasing ocean stratification over the past half-century. Nat. Clim. Chang..

[5] Schmidt H., Seitz S., Hassel E., Wolf H. (2018). The density-salinity relation of standard seawater. Ocean Sci..

[6] Qian Y., Zhao Y., Wu Q.L., Yang Y. (2018). Review of salinity measurement technology based on optical fiber sensor. Sens. Actuators B Chem..

[7] Demir O., Johnson J.T., Jezek K.C., Brogioni M., Macelloni G., Kaleschke L., Brucker L. (2022). Studies of sea-ice thickness and salinity retrieval using 0.5–2 GHz microwave radiometry. IEEE Trans. Geosci. Remote Sens..

[8] Cox R.A., Culkin F., Riley J.P. (1967). The electrical conductivity/chlorinity relationship in natural sea water. Deep Sea Res. Part I Oceanogr. Res. Pap..

[9] Dinnat E.P., Le Vine D.M., Boutin J., Meissner T., Lagerloef G. (2019). Remote sensing of sea surface salinity: Comparison of satellite and in situ observations and impact of retrieval parameters. Remote Sens..

[10] Zhou Y., Lang R.H., Dinnat E.P., Le Vine D.M. (2021). Seawater debye model function at L-band and its impact on salinity retrieval from aquarius satellite data. IEEE Geosci. Remote Sens. Lett..

[11] Liang H.L., Wang J., Zhang L.H., Liu J.C., Wang S.S. (2022). Review of optical fiber sensors for temperature, salinity, and pressure sensing and measurement in seawater. Sensors.

[12] Quan X., Fry E.S. (1995). Empirical equation for the index of refraction of seawater. Appl. Opt..

[13] Wang X., Wang J., Wang S.S., Liao Y.P. (2017). Fiber-optic salinity sensing with a panda-microfiber-based multimode interferometer. J. Light. Technol..

[14] Lin Z.T., Lv R.Q., Zhao Y., Zheng H.K., Wang X.X. (2021). High-sensitivity special open-cavity Mach-Zehnder structure for salinity measurement based on etched double-side hole fiber. Opt. Lett..

[15] Zheng H.K., Zhao Y., Lv R.Q., Lin Z.T., Wang X.X., Zhou Y.F., Hu X.G. (2021). Study on the temperature and salinity sensing characteristics of multifunctional reflective optical fiber probe. IEEE Trans. Instrum. Meas..

[16] Zhao Y., Wu Q.L., Zhang Y.N. (2019). Simultaneous measurement of salinity, temperature and pressure in seawater using optical fiber SPR sensor. Measurement.

[17] Siyu E., Zhang Y.N., Han B., Zheng W.L., Wu Q.L., Zheng H.K. (2020). Two-channel surface plasmon resonance sensor for simultaneous measurement of seawater salinity and temperature. IEEE Trans. Instrum. Meas..

[18] Yang M.S., Zhu Y.L., An R. (2021). Underwater fiber-optic salinity and pressure sensor based on surface plasmon resonance and multimode interference. Appl. Opt..

[19] Sun M.Y., Jiang H.T., Shi B., Zhou G.Y., Inyang H.I., Feng C.X. (2019). Development of FBG salinity sensor coated with lamellar polyimide and experimental study on salinity measurement of gravel aquifer. Measurement.

[20] Kumari C.R.U., Samiappan D., Kumar R., Sudhakar T. (2019). Development of a highly accurate and fast responsive salinity sensor based on Nuttall apodized fiber Bragg grating coated with hygroscopic polymer for ocean observation. Opt. Fiber Technol..

[21] Yang F., Sukhishvili S., Du H., Tian F. (2017). Marine salinity sensing using long-period fiber gratings enabled by stimuli-responsive polyelectrolyte multilayers. Sens. Actuators B Chem..

[22] Yang F., Hlushko R., Wu D., Sukhishvili S.A., Du H., Tian F. (2019). Ocean salinity sensing using long-period fiber gratings functionalized with layer-by-layer hydrogels. ACS Omega.

[23] Zhao S., Du C., Wang Q., Jia B., Zhang L., Cui L., Deng X., Chen S. (2023). A long period fiber grating seawater salinity sensor based on bend insensitive single mode fiber. Opt. Fiber Technol..

[24] Del Villar I. (2015). Ultrahigh-sensitivity sensors based on thin-film coated long period gratings with reduced diameter, in transition mode and near the dispersion turning point. Opt. Express.

[25] Cho Y., Ahmed F., Joe H.E., Yun H., Min B.K., Jun M.B.G. (2017). Fabrication of a screw-shaped long-period fiber grating for refractive index sensing. IEEE Photonics Technol. Lett..

[26] Dong X.R., Xie Z., Song Y.X., Yin K., Chu D.K., Duan J.A. (2017). High temperature-sensitivity sensor based on long period fiber grating inscribed with femtosecond laser transversal-scanning method. Chin. Opt. Lett..

[27] Colaco C., Caldas P., Del Villar I., Chibante R., Rego G. (2016). Arc-induced long-period fiber gratings in the dispersion turning points. J. Light. Technol..

[28] Xu X., Lu C.P., Jin X.R., Lv M.Y., Sun C.T., Yan Q., Zhang S., Wang J.B., Rui Z.J., Xiang Z.H. (2021). A vector bending sensor based on a core-offset long period fiber grating induced by an arc-discharge. IEEE Sens. J..

[29] Janczuk-Richter M., Dominik M., Razniecka E., Koba M., Mikulic P., Bock W.J., Los M., Smietana M., Niedziolka-Jonsson J. (2017). Long-period fiber grating sensor for detection of viruses. Sens. Actuators B Chem..

[30] Smietana M., Dominik M., Mikulic P., Bock W.J. (2018). Temperature and refractive index sensing with Al_2_O_3_-nanocoated long -period gratings working at dispersion turning point. Opt. Laser Technol..

[31] Del Villar I., Fuentes O., Chiavaioli F., Corres J.M., Matias I.R. (2018). Optimized strain long-period fiber grating (LPFG) sensors operating at the dispersion turning point. J. Light. Technol..

[32] Wang S.Y., Ma Y.W., Geng T., Sun C.T., Zhu H.J., Li X.Y., Yi Y., Zhang S., Sun W.M., Yuan L.B. (2020). Compact fiber strain sensor fabricated by a CO_2_ laser. Opt. Lett..

[33] Zhao J., Xu J.S., Wang C.X., Feng M., Zheng Y., Wang T., Wang Z. (2021). Critical grating period behavior of a sensitivity enhanced LPFG sensor written in a few-mode fiber. Opt. Commun..

[34] Pawlowicz R., Feistel R., McDougall T.J., Ridout P., Seitz S., Wolf H. (2016). Metrological challenges for measurements of key climatological observables Part 2: Oceanic salinity. Metrologia.

[35] Parra L., Viciano-Tudela S., Carrasco D., Sendra S., Lloret J. (2023). Low-cost microcontroller-based multiparametric probe for coastal area monitoring. Sensors.

[36] Le Vine D.M., Dinnat E.P. (2022). Sensitivity of wide bandwidth radiometer for remote sensing of ocean salinity. IEEE Trans. Geosci. Remote Sens..

[37] Zhao J., Zhao Y., Cai L. (2022). Hybrid fiber-optic sensor for seawater temperature and salinity simultaneous measurements. J. Light. Technol..

[38] Zhao Y., Wu Q.-L., Zhang Y.-N. (2018). Theoretical analysis of high-sensitive seawater temperature and salinity measurement based on C-type micro-structured fiber. Sens. Actuators B Chem..

[39] Yang X., Wang Z., Liu Y., Yao J. (2021). SPR sensor based on exposed core micro-structured optical fiber for salinity detection with temperature self-compensation. Opt. Mater. Express.

[40] Raghuwanshi S.K., Singh Y., Pandey P.S., Kumar R. (2021). Sensitivity analysis of HF etched uniformly thinned α-power refractive index profile fiber bragg grating sensor. IEEE Trans. Instrum. Meas..

[41] Dey T.K., Tombelli S., Biswas P., Giannetti A., Basumallick N., Baldini F., Bandyopadhyay S., Trono C. (2021). Analysis of the lowest order cladding mode of long period fiber gratings near turn around point. J. Light. Technol..

[42] Viveiros D., de Almeida J., Coelho L., Vasconcelos H., Maia J.M., Amorim V.A., Jorge P.A.S., Marques P.V.S. (2021). Turn around point long period fiber gratings with coupling to asymmetric cladding modes fabricated by a femtosecond laser and coated with titanium dioxide. J. Light. Technol..

[43] Du C., Wang Q., Zhao Y., Hu S. (2019). Ultrasensitive long-period gratings sensor works near dispersion turning point and mode transition region by optimally designing a photonic crystal fiber. Opt. Laser Technol..

[44] Jian P.S., Smela E. (2006). Modeling the performance of a long-period Bragg grating ambient-index sensor. Smart Mater. Struct..

[45] Shu X., Lin Z., Bennion I. (2002). Sensitivity characteristics of long-period fiber gratings. J. Light. Technol..

[46] Shu X., Zhu X., Jiang S., Shi W., Huang D. (1999). High sensitivity of dual resonant peaks of long-period fibre grating to surrounding refractive index changes. Electron. Lett..

[47] Jin L., Jin W., Ju J., Wang Y. (2010). Coupled local-mode theory for strongly modulated long period gratings. J. Light. Technol..

[48] Zhong X.Y., Wang Y.P., Liao C.R., Yin G.L., Zhou J.T., Wang G.J., Sun B., Tang J. (2014). Long period fiber gratings inscribed with an improved two-dimensional scanning technique. IEEE Photonics J..

[49] Smietana M., Koba M., Brzozowska E., Krogulski K., Nakonieczny J., Wachnicki L., Mikulic P., Godlewski M., Bock W.J. (2015). Label-free sensitivity of long-period gratings enhanced by atomic layer deposited TiO_2_ nanooverlays. Opt. Express.

[50] Del Villar I., Cruz J.L., Socorro A.B., Corres J.M., Matias I.R. (2016). Sensitivity optimization with cladding-etched long period fiber gratings at the dispersion turning point. Opt. Express.

